# Clinical, Molecular, and Genetic Characteristics of PAPA Syndrome: A Review

**DOI:** 10.2174/138920210793175921

**Published:** 2010-11

**Authors:** Elisabeth J Smith, Florence Allantaz, Lynda Bennett, Dongping Zhang, Xiaochong Gao, Geryl Wood, Daniel L Kastner, Marilynn Punaro, Ivona Aksentijevich, Virginia Pascual, Carol A Wise

**Affiliations:** 1 Sarah M. and Charles E. Seay Center for Musculoskeletal Research, Scottish Rite Hospital for Children, Dallas, Texas 75219; 2 Garvan Institute of Medical Research, Sydney, NSW 2027, Australia; 3 Baylor Institute for Immunology Research, Dallas, Texas 75204; 4 Laboratory of Clinical Investigation, National Institute of Arthritis and Musculoskeletal and Skin Diseases, Bethesda, Maryland 20892, USA

**Keywords:** Auto-inflammatory disease, PAPA syndrome, *PSTPIP1*, *CD2BP1*, PTP-PEST, pyrin, neutrophils, microarray transcript profiling, anakinra, IL-1β.

## Abstract

PAPA syndrome (Pyogenic Arthritis, Pyoderma gangrenosum, and Acne) is an autosomal dominant, hereditary auto-inflammatory disease arising from mutations in the *PSTPIP1*/*CD2BP1 *gene on chromosome 15q. These mutations produce a hyper-phosphorylated PSTPIP1 protein and alter its participation in activation of the “inflammasome” involved in interleukin-1 (IL-1β) production. Overproduction of IL-1β is a clear molecular feature of PAPA syndrome. Ongoing research is implicating other biochemical pathways that may be relevant to the distinct pyogenic inflammation of the skin and joints characteristic of this disease. This review summarizes the recent and rapidly accumulating knowledge on these molecular aspects of PAPA syndrome and related disorders.

## INTRODUCTION

The auto-inflammatory disorders bear resemblance to classic autoimmune diseases such as systemic lupus in that they present with seemingly unprovoked inflammation but, unlike most autoimmune diseases, high-titer auto-antibodies or antigenic-specific T lymphocytes are not found [1-3]. Considered broadly, this disease class is characterized by abnormalities in the innate immune system. Familial Mediterranean Fever (FMF), a recessive disorder caused by mutations in the gene encoding the pyrin protein, is the founding member of this disease class [3] that is marked by episodic inflammation of serosal or synovial tissue, fever, and occasional lesions of the skin. Many disorders are now recognized as auto-inflammatory. Some, like FMF, display simple Mendelian inheritance and are caused by mutations in single genes [2-4]. Examples include TNF receptor-associated periodic syndrome (TRAPS) [2], hyperimmunoglobulinemia D with periodic fever syndrome (HIDS) [5, 6], familial cold auto-inflammatory syndrome (FCAS)/Muckle-Wells syndrome (MWS), neonatal onset multisystem inflammatory disease (NOMID) [7], deficiency of the interleukin-1-receptor antagonist (DIRA) [8, 9]; the granulomatous diseases such as Blau syndrome [10], early onset sarcoidosis [11], and chronic granulomatous disease (CGD) [12]; and the pyogenic disorders Majeed syndrome [13] and PAPA syndrome [14]. Auto-inflammatory underpinnings are now also recognized in common, less heritable conditions including Crohn’s disease and systemic-onset juvenile idiopathic arthritis (SoJIA) [15-17]. Over the past decade our understanding of both the clinical characteristics and molecular pathogenesis of the auto-inflammatory diseases has expanded dramatically (for an extensive review of this disease class please see reference [3]). Accordingly, a new classification system was recently proposed that defines auto-inflammatory disease by one of six categories according to its underlying molecular pathology [3]. The chronicle of PAPA syndrome, a relatively recent addition and one of the more clinically dramatic members of the auto-inflammatory disease family, is a relevant illustration of diagnostic, molecular, and clinical advances in the field. In this review we summarize the ongoing elucidation of PAPA syndrome pathogenesis, with particular regard to the impact of molecular discovery on the diagnosis and management of affected patients and families.

## CLINICAL CHARACTERISTICS OF PAPA SYNDROME

**Fig. (1):**
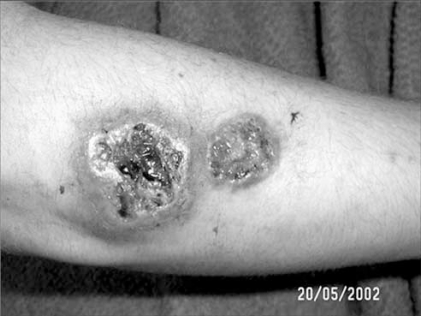
Pyoderma gangrenosum in a child with PAPA syndrome.

PAPA syndrome (OMIM #604416) was described as a heritable disease in an extended family in 1997 [14]. We now appreciate that PAPA syndrome was in fact first reported in a single male patient described with “streaking leukocyte factor”, arthritis, and pyoderma gangrenosum in 1975 [18]. “Streaking leukocyte factor” referred to a partially purified, ~160 kd component of this patient’s serum that enhanced the random migration of normal mononuclear cells and neutrophils in laboratory studies. (The identity of this “factor” is unresolved.) Both reports described joint inflammation as generally responsive to high dose corticosteroids. A second extended family was described in 2000 as “familial recurrent arthritis” [19]. Additional families and cases are now reported (described later in this review). Distinguishing features of PAPA syndrome include early onset, florid, and painful flares of recurrent sterile arthritis involving a prominent neutrophilic infiltrate, and autosomal dominant inheritance. Skin involvement is more variable and may present as ulcerations, frank pyoderma gangrenosum, or severe cystic acne (Fig. **1**). Standard laboratory findings typically reflect systemic inflammation but are otherwise non-diagnostic; however elevated production of interleukin-1 beta (IL-1β) and tumor necrosis factor (TNFα) in peripheral blood leukocytes has been reported in more recent literature [20-22]. Symptoms persist into adulthood, with a natural history of significant joint destruction. Anecdotally, some individuals report significant psycho-social impairment due to physical disability, steroid-induced Cushingoid appearance, and permanent, wide-spread cutaneous scarring.

## PSTPIP1 / CD2BP1

Positional cloning methods subsequently identified co-segregating missense mutations in the proline-serine-threonine phosphatase-interacting protein 1 gene (*PSTPIP1*, also known as CD2 binding protein 1 (*CD2BP1*)) in the original family that defined the syndrome and a second extended family (originally described with “familial recurrent arthritis”) [23-24]. Other PAPA syndrome patients with either of these *PSTPIP1* mutations have been reported more recently [25, 26]. Specifically, missense mutations (c.904G>A) or (c.964G>C) occur in exons 10 and 11, creating A230T or E250Q variants, respectively, in affected individuals. The observation of the same mutation in seemingly unrelated families suggest either a founder effect, particularly as these were each observed in cases within the same ethnic group, i.e. European descent, or a mutational hotspot. Otherwise, two additional missense mutations are reported, both in exon 11 and creating E250K or D260N amino acid changes. This genotype and phenotype information is documented and continuously updated for PAPA syndrome and the other auto-inflammatory diseases within the INFEVERS database, a web-based resource (please see http://fmf.igh.cnrs.fr/ISSAID/infevers/) [17]. It is interesting that other putative cases of PAPA syndrome have proven negative for *PSTPIP1* coding or splicing mutations (Table **1**) [our unpublished data and reference 27]. This suggests the possibility that alterations in other gene(s) could evoke features of PAPA syndrome. Notably, also, cases with isolated pyoderma gangrenosum or Crohn’s disease-associated pyoderma gangrenosum, have thus far proven negative for *PSTPIP1* mutations. However, recently a CCTG repeat in the *PSTPIP1* promoter of patients with Crohn’s disease or aseptic abscesses syndrome was identified [28] that may play a pathogenic role in these diseases. *CD2BP1 *(*PSTPIP1*) and *CARD15 *mutations are not associated with pyoderma gangrenosum in patients with inflammatory bowel disease (Table **1**) [29]. Further study is required to explain *PSTPIP1* mutation-negative cases of putative PAPA syndrome. In the meantime, the low complexity of disease-associated mutations in *PSTPIP1* simplifies diagnostic screening for patients with a tentative diagnosis of PAPA syndrome, and a DNA sequence-based test for *PSTPIP1* coding mutations is now commercially available. It is worth noting that the PAPA-associated mutations presently identified are highly predictive, as reflected by the essentially complete disease penetrance they confer, and thus provide useful information in the context of genetic counseling for families.

**Fig. (2):**
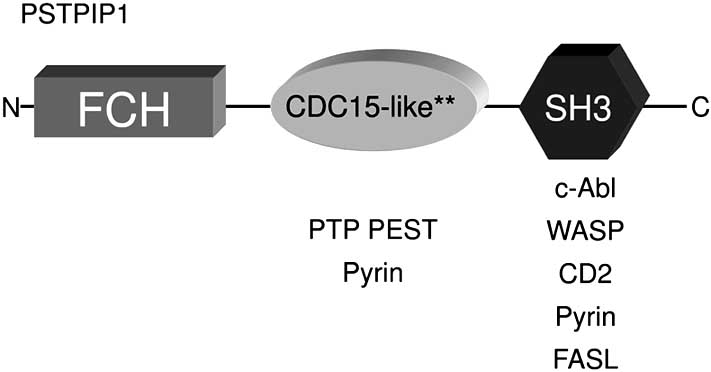
*PSTPIP1* protein schematic. FCH, coiled-coil, and SH3 domains are shown. Asterisks denote the relative positions of the A230T and E250Q mutations. Interacting proteins are shown below single domains for simplicity. It should be noted that some *in vitro* studies indicate more complex binding patterns than depicted here.

Interestingly, there is a homolog of *PSTPIP1*, named proline-serine-threonine-phosphatase-interacting protein 2 (*PSTPIP2*), encoded on chromosome 18 in both humans and the mouse. PSTPIP1 and PSTPIP2 predicted proteins share approximately 41% amino acid identity, but PSTPIP2 is lacking the terminal src-homology 3 (SH3) domain found in PSTPIP1 (Fig. **2**) [30]. At present it is unclear if PSTPIP1 and PSTPIP2 are completely distinct, or if they participate in overlapping biochemical pathways. Both proteins possess Fer-CIP4 and coiled-coil domains involved in binding with PEST-type phosphatases. Fer-CIP4 domains also bind phosphatidyl inositol (4, 5) bisphosphonate (PI(4,5)P2) on artificial liposomes with high affinity, inducing membrane tubulation and suggesting that both could participate in coupling membrane deformation to cytoskeletal reorganization (described in more detail below) [31]. Several lines of evidence link *PSTPIP2* with yet another auto-inflammatory condition. In the mouse, spontaneous recessive mutations in *PSTPIP2* produce a phenotype most closely resembling human chronic multifocal osteomyelitis (CRMO), another auto-inflammatory disease of bone and skin. Human PSTPIP1 is encoded on chromosome 18q21.3-22 and overlaps a susceptibility locus for CRMO [32]. Based on these observations, *PSTPIP2* could be an attractive candidate gene for cases with PAPA-like features that are negative for *PSTPIP1* mutations, however, no mutations have been identified. Further functional studies, particularly utilizing *PSTPIP2* mouse mutants, will provide insight into the biochemical, and possibly clinical relationship of these two proteins.

## PSTPIP1 BINDING PROTEINS

PSTPIP1 is a cytoskeletal adaptor protein that was originally identified in the mouse through its interaction with PEST (rich in proline (P), glutamic acid (E), serine (S), and threonine (T) residues)-type protein tyrosine phosphatase (PTP-PEST, also known as PTPN12) [33]. The human homolog, called CD2BP1, was identified near the same time by interaction with the T cell surface protein CD2 [34]. The gene and protein are now generally cited as “PSTPIP1*”*. The fact that PAPA syndrome mutations clustered in the coiled-coil region of the protein immediately cast suspicion that the PTP-PEST interaction mediated through this domain was central to pathology. Indeed, the E250Q and A230T variants of PSTPIP1 were shown to severely abrogate binding to PTP-PEST in yeast two hybrid and co-immunoprecipitation experiments [21, 24]. At least one consequence of this is hyperphosphorylation of PSTPIP1 itself. Tryptic peptide mapping identified tyrosine 344 as a primary phosphorylation site of PSTPIP1. Experiments using a combination of co-transfection, immunoprecipitation and anti-phosphotyrosine antibody western blots have shown that binding of PTP-PEST to PSTPIP1 is essential for its dephosphorylation [21, 35, 36]. Interestingly, PTP-PEST^−/−^ fibroblasts contain hyperphosphorylated PSTPIP1 and a defect in cytokinesis [35].

PSTPIP1 is highly expressed in hematopoietic cells, and the protein interacts with other immune-related proteins in addition to CD2 and PTP-PEST. These include Wiskott Aldrich Syndrome protein (WASP), c-Abl kinase, and Fas ligand (FasL) [36-38]. Cumulative evidence suggests that each of these proteins acts as a “substrate”, binding primarily via the PSTPIP1 SH3 domain (Fig. **2**) to be delivered to PTP-PEST (or homolog) for de-phosphorylation [39-41]. It is not obvious whether A230T or E250Q mutations directly alter these interactions, or may mediate effects due to hyperphosphorylation of the “substrate” or of PSTPIP1 itself. For example, the PSTPIP1/WASP interaction is phosphorylation-dependent, and disease mutations consequently predict reduced interaction *in vivo*. Thus WASP-mediated cytoskeletal reorganization events are implicated in PAPA pathogenesis via post-translational mechanisms [24, 36]. In the case of FasL, it is clear that this protein forms a ternary complex with PSTPIP1/PTP-PEST, and that PSTPIP1 mediates its sequestration away from the cell surface and into intracellular secretory lysosomes [38, 39]. This suggests a general inhibitory effect on cytotoxic T cell functioning. Further study will elucidate the effects of PAPA causative mutations on these interactions and their relationship to manifestations of the disease (see more discussion later in this review). Taken altogether, current knowledge suggests multiple functions for a PSTPIP1/PTP-PEST complex in hematopoietic cells that may have direct consequences for immune cell adhesion, invasion, and migration [36-46]. It is worth noting that PSTPIP1 also interacts with other PTP-PEST homologs such as PTP hematopoietic stem cell factor (HSCF) [33], suggesting possible roles in functions such as lymphocyte activation, antigen processing, granule exocytosis, phagocytosis and apoptosis, as the PEST family phosphatases have been implicated in these processes [47].

PSTPIP1 also binds itself. Western blots of patient and control monocyte lysates run on native gels clearly revealed a major band twice the size of PSTPIP1 monomer when probed with an anti-PSTPIP1 polyclonal antibody. Weaker bands possibly corresponding to PSTPIP1 trimers or tetramers were also observed [48]. *In silico* modeling predicted that the dimerized protein assumes a gently curved structure that is not altered by disease-causing mutations. Other work has suggested the PSTPIP1 exists as a homotrimer [49]. Either way, evidence suggests that disease-causing mutations in PSTPIP1 do not alter its self-binding capacity.

## PSTPIP1 AND PYRIN

As noted earlier, mutations in the pyrin protein are responsible for FMF, a disease that shows some clinical similarities to PAPA syndrome, including a neutrophil-rich sterile infiltrate of the joints, neutrophilic dermatoses, and elevated production of IL-1 by peripheral blood leukocytes [50-52]. The relationship of these two diseases was clarified by the discovery of molecular overlap, when a pyrin bait identified PSTPIP1 in yeast two hybrid screens of a monocyte library. Subsequent work confirmed the direct interaction of pyrin and PSTPIP1. Furthermore, hyperphosphorylated variants of PSTPIP1 (e.g. containing disease-causing mutations) displayed a higher affinity for pyrin than wild type forms [21]. This discovery was a watershed moment, as it linked the two diseases within the same previously described pathway and suggested a perturbation that could be targeted therapeutically. Specifically, pyrin has been proposed to activate IL-1β production via a complex known as the “inflammasome”. Although the composition of inflammasomes may vary, for the purposes of this discussion we focus on a canonical complex of three proteins: NLRP3/ASC/Caspase-1. NLRP3, also called cryopyrin, contains a pyrin-like domain through which it interacts with apoptosis-associated speck-like protein with a caspase recruitment domain (ASC). ASC in turn interacts with caspase 1 that catalytically cleaves pro-IL-1β into its active form. Upon activation, the inflammasome complex cleaves pro-IL-1β to produce mature IL-1β that is subsequently secreted, perhaps through a pathway of lysosome exocytosis [53, 54]. Mutations in *NLRP3 *itself cause the auto-inflammatory syndromes FCAS/MWS/NOMID described earlier. How pyrin participates in this or other inflamma-somes is the subject of ongoing investigations. However, the experiments of Shoham *et al.* clearly demonstrated overproduction of IL-1β in the presence of PAPA-associated mutations relative to wild type in a heterologous, reconstituted system, as well as elaboration of IL-1β in lipopolysaccharide (LPS)-stimulated monocytes of a PAPA syndrome patient [21]. IL-1β precursor transcripts are also highly over-expressed in circulating neutrophils from PAPA syndrome patients as compared to healthy control individuals by transcript profiling (our unpublished observations).

**Fig. (3):**
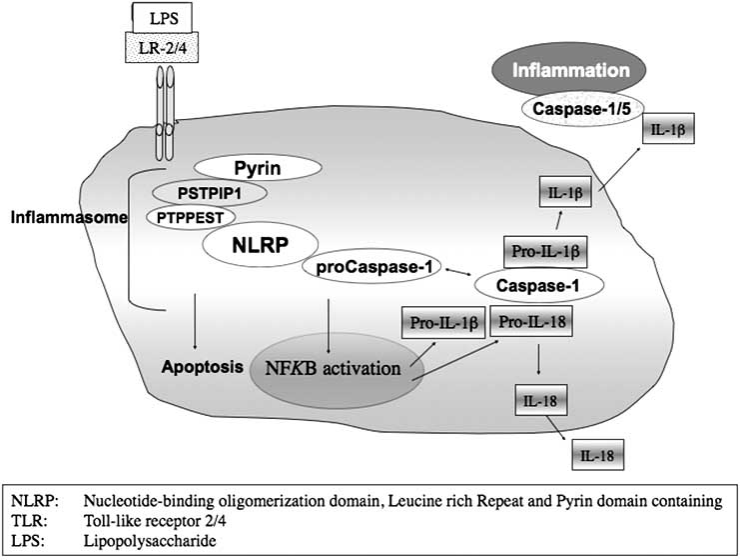
Schematic depiction of the “inflammasome.” IL-1β and IL-18 are substrates for caspase.

More recent studies have suggested an alternative mechanism in which the balance of interaction between PSTPIP1, pyrin and ASC is altered by PSTPIP1 variants E250Q and A230T in monocytes, leading to activation of the so-called “pyroptosome” that leads to cell death and release of cytokines such as IL-1β [49, 55]. PSTPIP1 associates with microtubules in native and transfected cells and does not associate with the ASC compartment in the absence of pyrin [48, 49]. Pyrin modulates PSTPIP1 intracellular distribution and is apparently necessary for PSTPIP1-mediated regulation of the ASC pyroptosome. Importantly, either “inflammasome” or “pyroptosome” mechanisms predict overproduction of IL-1β in the presence of PAPA causal mutations (Fig. **3**). It is worth noting that while such disease-causing mutations affect pyrin-mediated pathways, the reverse is apparently not the case, i.e. FMF causal mutations do not affect binding to PSTPIP1. This is generally explained by the fact that such mutations almost always occur in pyrin outside the PSTPIP1 binding site, a B box domain that mediates pyrin autoinhibition in the absence of PSTPIP1 [56]. Two B box variants, P369S and R408Q, may play a role in FMF susceptibility but do not appear to alter PSTPIP1 interaction [56].

## PAPA SYNDROME AS AN “INFLAMMASOMOPATHY”

Studies described above firmly established PAPA syndrome as a member of the auto-inflammatory disease family, and more specifically, an “inflammasomopathy” according to the new classification system proposed by Masters *et al.* [3]. IL-1β hence emerged as a prime therapeutic target for PAPA syndrome. Fortunately, biological agents that target this cytokine cascade are available. Anakinra, a recombinant IL-1 receptor antagonist that is administered by daily parenteral injection, has proven to be effective in controlling flares for PAPA syndrome patients [57, 58]. Indeed, in our experience, this treatment is very effective in resolving joint inflammation altogether for some patients.

Pharmaceuticals that target downstream events in IL-1β signaling may also prove effective in some patients. For example, IL-1β is a potent inducer of TNFα, a major stimulant of apoptosis and inflammation, and an anti-TNFα monoclonal antibody, infliximab, has shown promising results in controlling symptoms of PAPA syndrome, including dramatic resolution of severe pyoderma gangrenosum in one patient [59-61]. In contrast, cystic acne, the second cutaneous symptom of PAPA syndrome, does not seem as responsive to IL1β and TNFα blockade (unpublished observations), raising interesting questions about the pathogenesis of each of the manifestations of the disease. More clinical experience with PAPA syndrome should help to clarify and prioritize best treatment options in the face of varying symptoms in specific target organs.

## INSIGHTS INTO DISEASE PATHWAYS

Il-1β and TNF-α-targeted therapies (so-called “biologics”) have provided a generally superior alternative to high-dose corticosteroids in relieving inflammatory flares of PAPA syndrome. However, in collective experience these biologics have not been consistently effective in all cases and do not necessarily hasten remission of all the disease manifestations. This may not be surprising in view of the evidence that PSTPIP1 likely functions in multiple biochemical pathways in several immune-related cells (T cells, neutrophils, monocyte-derived cells, NK cells). IL-1β/TNF-α antagonists suppress innate immune mechanisms fairly broadly and convey significant risk of infection, a particularly troublesome problem when faced with managing simultaneous open skin lesions. Clearly, more detailed studies of PSTPIP1-mediated pathways are needed to better understand disease mechanisms in relevant tissues (i.e. joints and skin) and thereby inform the development of more specific therapies. Genetic studies in appropriate animal models will likely be invaluable to these efforts. Meanwhile studies in patient cells have provided some initial insights. A recent study of macrophages cultured from monocytes of four PAPA syndrome patients (with confirmed A230T or E250Q mutations) revealed deficiencies in chemotaxis and invasive migration. In contrast, impairment of these functions was not observed in similar tests of patient T cells. In addition, PAPA syndrome macrophages formed significantly fewer podosomes than controls, and podosome structures were altered in that they contained increased numbers of focal complexes [62]. This is consistent with expression data revealing increased transcript levels in PAPA individuals for proteins involved in focal adhesion complexes (e.g. gelsolin, integrin-beta3, talin, PTP-PEST) [our unpublished observations]. The authors also noted that the dismorphology of macrophages observed in PAPA syndrome is reminiscent of that seen in Wiskott-Aldrich Syndrome, suggesting a common PSTPIP1/WASP pathway is disrupted in both diseases, at least in monocytes/macrophages. It was further hypothesized that the dramatic recruitment of neutrophils during PAPA syndrome flares is a response to signaling from macrophages retained in affected sites [62]. Certainly prior studies of patient peripheral blood leukocytes and serum suggest a gain-of-function, pro-inflammatory state in these tissues, as evidenced by overproduction of IL-1β and TNF-α, and the original ~160 kd “streaking leukocyte factor” that stimulated random migration of normal neutrophils and mononuclear cells *in vitro *[18].

Although cutaneous involvement is variable in PAPA syndrome it is a particularly troubling feature of the disease, not only in terms of clinical management but also cosmesis. Regarding the latter, some PAPA syndrome patients become quite reclusive because of concerns about their appearance. Pyoderma gangrenosum (and cystic acne) are characterized by neutrophil infiltration; given the strong *PSTPIP1* expression in neutrophils we hypothesize that this is at least in part secondary to an intrinsic mechanism in these cells. Recent cell transfection studies by Cooper *et al.* found that PSTPIP1 localizes to the neutrophil uropod and alters motility and endocytosis. However, A230T and E250Q mutants did not alter these functions, and thus, the role of disease mutations in neutrophil behavior is unresolved [42]. As noted, PSTPIP1 pathways in non-myeloid cells such as T cells may also factor in PAPA syndrome.

**Fig. (4):**
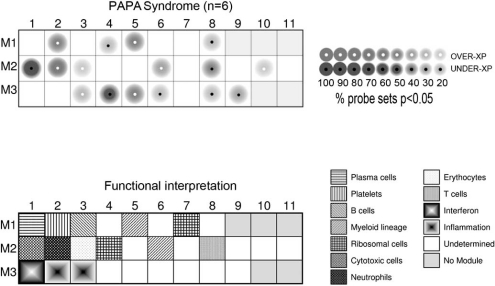
Modular analyses of PAPA blood gene expression reveals over-expression of neutrophil and platelet-related genes. mRNA expression level differences in PBMCs between PAPA patients and age- and sex-matched healthy controls were obtained by statistical group comparison. We further performed modular analyses as described in [67, 68]. Briefly, genes are assigned *a priori* to particular functional units (i. e. modules) as derived from extensive analyses of expression in PBMCs. Statistical comparisons are then made between disease groups and healthy groups on a module-by-module basis, where the proportion of under- or over-expressed genes in the module is depicted in the cartoon by differences in color and intensity. This approach simplifies analysis and produces a distinct “fingerprint” of disease. A complete list of differentially expressed gene transcripts in PAPA syndrome individuals versus controls is available upon request.

Global gene expression analysis has proved to be a useful tool for dissecting underlying pathways in blood of patients with various inflammatory diseases [63-67]. This approach has been particularly powerful as a molecular diagnostic tool for certain complex diseases with relatively non-specific features, such as SoJIA. Like PAPA syndrome, SoJIA is an auto-inflammatory disease marked by overproduction of IL-1β. Gene expression in blood of SoJIA and PAPA shows extensive overlaps (our unpublished observations), yet So-JIA predictive genes can distinguish it from PAPA [66]. Our preliminary observations of gene expression in peripheral blood mononuclear cells of PAPA patients using modular analyses have revealed strongest up-regulation of genes expressed in low-density neutrophils as well as in platelets and myeloid cells (Modules 2.1, 1.2, and 1.5, respectively, Fig. **4**). As examples, over-expressed neutrophil-related genes included defensin A4 (DEFA4), peptidyl arginine deiminase 14 (PAD14), lipocalin 2 (LCN2), and forkhead box O3A (FOXO3A) among others. In addition, genes involved in cytotoxic cell functions were strongly under-expressed relative to controls (Fig. **4**). Over-expression of neutrophil-related genes in PBMC fractions has been observed in other inflammatory diseases such as SLE and SoJIA and may indicate over-representation of neutrophil precursors in PAPA blood, although further study is required to determine this.

## SUMMARY

The convergence of biochemical, cell biological, molecular genetic, and clinical studies in various experimental systems has brought PAPA syndrome (and afflicted patients) out of obscurity, clearly revealing causation and pathways to therapy. It has also raised intriguing new questions. The classification of PAPA syndrome as an inflammasomopathy implies a pro-inflammatory state with heightened response to environmental triggers. If so, are patients protected from generalized infections? This is difficult to determine from limited patient studies but an interesting concept to explore, perhaps in animal models. The disease pathogenesis of PAPA syndrome may be explained by the mutations in PSTPIP1 that alter its interaction with pyrin and the inflammasome. Alternatively, is pyroptosome formation and cell death relevant in vivo, for patients with PAPA syndrome? Either way, an apparent consequence of this is an overproduction of active IL-1β. In this scenario, are there specific microbial pathogens, other stimuli, or simply trauma that provoke an exuberant response and consequent flares? It will be particularly important to investigate these mechanisms not only for sterile compartments (i.e. joints) but also in the skin, where interactions with comensal and pathogenic microbes abound [3]. Given the localization of PSTPIP1 along microtubules and its association with pyrin, why is colchicine ineffective in treating for PAPA syndrome patients? As PSTPIP1 is a cytoskeletal protein, could it regulate IL1β secretion rather than activation? Therapies targeting IL1β also lack efficacy in some individuals. Could this be explained by upregulation of interleukin 18, which is also activated by the inflammasome, in PAPA patients? Finally, as with virtually all Mendelian disorders the variability in disease severity even within single families is baffling. The emerging era of whole genome sequencing may provide the opportunity to identify genetic modifiers that will truly enlighten interventions for episodic diseases such as PAPA syndrome.

## Figures and Tables

**Table 1:** **Summary of *PSTPIP1* Mutation Screening for Cases of Putative PAPA Syndrome.** Cases were screened in the order given. The last three cases were accepted for screening based on the presence of three or more features of PAPA syndrome. *Exons 10 and 11 only were screened.

Subject	Country of Origin	Sex	Family History	Onset	Clinical Characteristics	PSTPIP1 Mutation
1	Spain	M	affected brother, mother	Child	arthritis; psoriasis; pyoderma gangrenosum	A230T (confirmed in affected brother)
2	Italy	M	mother mildly affected	Child	“consistent with PAPA syndrome”	Negative
3	USA	M	negative	Adult	inflammations around surgical incisions-pyoderma gangrenosum? Subsequent severe bleeding. Responsive to cortico-steroids	Negative
4	USA	F	multiple individuals with reported arthritis, fibromyalgia, diabetes mellitus	7	severe pauci-articular corticosteroid responsive arthritis; recurrent destructive pyoderma gangrenosumat age 11; recent dep venous thrombosis, pulmonary hypertension and congestive heart failure	None detected in screens of exon 10 or 11
5	Canada	M	undetermined; father’s history incomplete	1	Recurrent sterile pyogenic arthritis in knee; fever, synovial expansion into muscle above and below knee	A230T
6	New Zealand	M	positive with apparent dominant inheritance	1.5	Recurrent pyogenic arthritis	E250Q (confirmed in affected parent)
7	USA	M	mother with positive ANA and acne	14	arthritis in fingers, toes, and large joints; severe acne; ulcerated lesions bilaterally on hands, forearms, elbows described as pyoderma gangrenosum; aphthous ulcers; negative laboratory findings	None detected in screens of exons 10 and 11
8	France (Toulouse)	M	negative	?	recurrent arthritis of knees; painful involvement of chest, lower back, and sacrum regions; acute episodic abdominal pain and fever; severe cystic acne; recurrent pyoderma of axillae, groin, and face	Negative
